# Comparison of two postoperative complication grading systems after treatment of stifle and shoulder instability in 68 dogs

**DOI:** 10.1111/vsu.13893

**Published:** 2022-10-03

**Authors:** Faolain M. Barrett, Jason A. Bleedorn, Kyle D. Hutcheson, Bryan T. Torres, Derek B. Fox

**Affiliations:** ^1^ Department of Surgical Sciences, School of Veterinary Medicine University of Wisconsin‐Madison Madison Wisconsin USA; ^2^ Department of Veterinary Medicine and Surgery, College of Veterinary Medicine University of Missouri Columbia Missouri USA

## Abstract

**Objective:**

(1) To adapt and apply the Clavien–Dindo (aCD) postoperative complication grading system to dogs experiencing complications following a single orthopedic procedure. (2) To compare the reliability of the Clavien–Dindo system to the Cook complication grading system.

**Study design:**

Retrospective study.

**Sample population:**

Sixty‐eight client‐owned dogs.

**Methods:**

Scenarios derived from complications following TightRope stabilization of the stifle and shoulder were graded by four ACVS‐boarded surgeons using two systems; the Cook 3‐point scale and the aCD 5‐point scale. Because the aCD system distinguishes complications from outcomes (“sequelae” or “failure to cure”), two data sets were created: one with (*n* = 76) and without (*n* = 67) inclusion of “sequelae” and “failure to cure” cases. Interobserver reliability was evaluated using intraclass correlation coefficient (ICC) calculations.

**Results:**

Seventy‐six scenarios from 68 records were evaluated. The ICC of the aCD system was 0.620 consistent with moderate reliability. The reliability of the Cook system was good, with an ICC of 0.848. Exclusion of cases with “sequelae” or “failure to cure” resulted in excellent reliability of the aCD system (ICC = 0.975) and good reliability of the Cook systems (ICC = 0.857).

**Conclusion:**

The aCD grading system was less reliable than the Cook system when evaluating all cases but more reliable when evaluating cases of complications excluding “sequelae” and “failures to cure”.

**Clinical significance:**

The Cook grading system is reliably good in grading postoperative complications in dogs. The aCD system can also be used to assess postoperative complications with excellent reliability but is less reliable when distinguishing complications from other postoperative outcomes.

## 1 INTRODUCTION

Postoperative complications can be prevented, but not eliminated, justifying accurate reporting of safety and efficacy of surgical procedures. Such assessment would ideally rely on a universal classification and reporting process among surgeons.^1^ Authors of a systematic review of surgical treatment modalities for cranial cruciate ligament disease describe the difficulty in comparing studies due to the variability in methodologies of reporting postoperative incidences and outcome measurements.^2^ A standardized method of grading complications in veterinary surgery has been proposed by Cook et al.^3^ but may be more open to individual interpretation because of its reliance on subjective terminology such as “minor”, “major”, and “catastrophic”. Further, the reliability of this method between different individuals has never been examined.

The Clavien–Dindo postoperative complication classification system is well established in human surgery for its simplicity, reliability, and comprehensiveness.^4^, ^5^, ^6^, ^7^ Originally validated for application in general surgery, the Clavien–Dindo system has been successfully adapted for use in specific orthopedic procedures in people with similarly good reliability.^8^ Recently, the system has been used to describe postoperative complications in veterinary general surgery applications with success.^9^, ^10^, ^11^, ^12^ Nicholson et al., assessed the agreement between 8 institutions using a modified Clavien–Dindo system for grading complications following gastrointestinal surgeries in small animals and documented that 73% of cases reviewed showed moderate or good agreement.^12^

Both the Clavien–Dindo and Cook systems of grading complications focus on the therapy used to treat the given complication. However, in contrast to the Cook system, the Clavien–Dindo system distinguishes complications from two specific outcomes: a variety of surgical “sequelae” or “failures to cure”.^5^ Used here, a “sequela” is defined as an event inherent and inevitable to the surgical procedure and “failure to cure” refers to when the original goal of the surgery has not been met in the absence of complications.^5^, ^6^ The Cook system may be seen to include sequelae as minor complications and does not distinguish failures to cure specifically.^3^ Per Clavien–Dindo, the categories of true complications are stratified on a numeric 5‐point scale in order of increasing invasiveness of therapeutic interventions and worsening prognosis.^13^ The benefits of utilizing a system like the Clavien–Dindo scheme are to avoid over‐reporting complications in cases experiencing sequelae or a failure to cure and to provide greater complication grading resolution via a 5‐point scale versus the three main categories of the Cook system. Considering the absence of research assessing the reliability of the Cook system, and the growing use of the Clavien–Dindo system in both human and veterinary surgery, the objectives of this study were to adapt and apply the Clavien–Dindo system of grading postoperative complications to a population of dogs and compare its reliability to that of the Cook system. We hypothesized that the Clavien–Dindo system would demonstrate equivalent reliability between assessors when compared to the Cook classification system. 

## 2 MATERIALS AND METHODS

### 2.1 Case selection

Cases experiencing potential complications following the performance of a singular orthopedic technique used to treat either stifle or shoulder instability (TightRope: Arthrex Vet Systems, Naples, Florida) in dogs were examined. For the purposes of this study, the term “complication” referred to any postoperative deviation from an uneventful recovery that was a concern to the client, referring veterinarian or the attending surgeon. Medical records from the University of Missouri's databank was searched from the years 2007 to 2020 for cases in which a complication related to the previous performance of a TightRope stabilization for either stifle or shoulder instability was documented. Inclusion criteria included the complete reporting of the case history in the medical record, access to diagnostic imaging and a thorough description of the nature of the presenting complication. From these records, 76 cases were selected for complication grading as case scenarios. A scenario represented a single joint that underwent a TightRope stabilization that was later identified as possessing a potential complication. Thus, single dog undergoing separate, nonsimultaneous procedures on different joints could represent multiple case scenarios. The records for each case were reviewed by a single investigator (FB) and summarized as case scenarios that included the patient signalment, presenting complaint, diagnosis, procedures performed, associated diagnostic images and postoperative assessments for every visit associated with the primary surgical problem. Postoperative assessments consisted of follow‐up examinations and diagnostics, as well as follow‐up owner communications. A data set of 76 case scenarios obtained from 76 different surgeries performed in 68 dogs represented the study population. Within these case scenarios, a singular event was highlighted to be graded so both systems would be used to evaluate a complication at a given point in time, and not the entire case history. Two examples of case summaries are detailed below.

**FIGURE 1:**
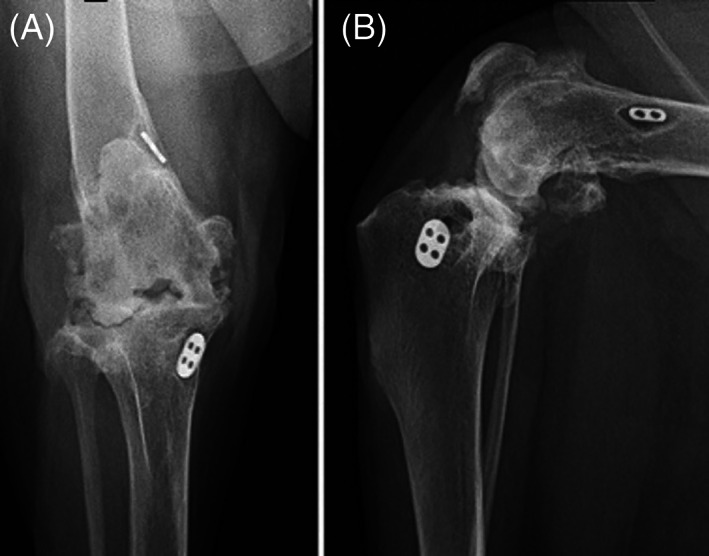
Radiographs of a left stifle TightRope implant 4 months after initial surgery: (A) craniocaudal view, (B) mediolateral view. The radiographs reveal marked bone lysis around the implant, joint space collapse, pronounced periarticular osteophytic proliferation, and severe bone remodeling. All assessors graded a grade 4 complication using the aCD system and a major complication using the Cook system.

Example 1: Male castrated, mixed breed dog – left cranial cruciate ligament rupture with associated stifle instability treated with TightRope stabilization; 1‐month post‐surgery the patient became lame and developed a draining tract from the incision site. Clinical signs resolved after antibiotic therapy. Four months postoperatively, the patient began to lick the original incision site. Radiographs revealed bone lysis around the implant, joint space collapse, pronounced periarticular osteophytic proliferation, joint space narrowing and remodeling (Figure 1). The implant was surgically removed along with necrotic tissue. The incision was left open to allow drainage and the patient stayed in hospital for 16 days when the wound was closed following culture and sensitivity results. The cultures revealed six different resistant Staphylococcus species. The patient was discharged with multiple antibiotics. No further follow‐up.

**FIGURE 2:**
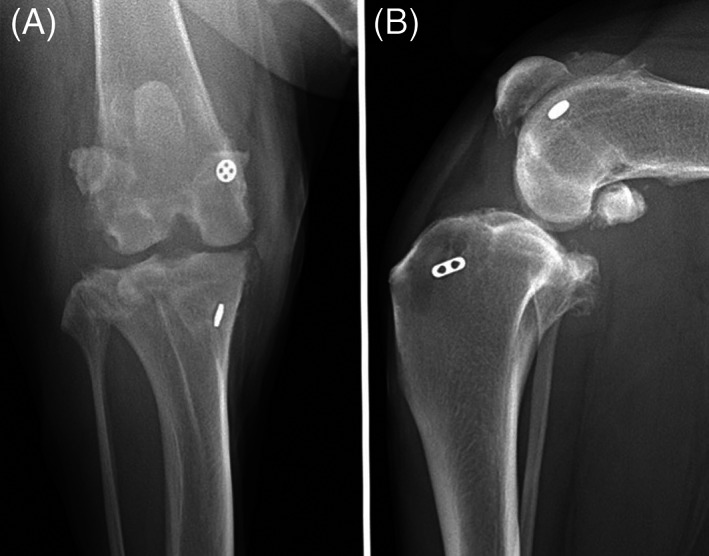
Radiographs of a left stifle TightRope implant 2 years, 11 months following initial surgery: (A) craniocaudal view, (B) mediolateral view. The radiographs reveal static implant positioning with periarticular osteophytic proliferation, joint effusion and mild bone tunnel widening. All assessors graded a grade 3 complication using the aCD system and a major complication using the Cook system.

Example 2: Male castrated, Labrador retriever ‐ left cranial cruciate ligament rupture with associated stifle instability treated with TightRope stabilization; Thirteen months post‐surgery, the patient presented for left hindlimb lameness. Orthopedic examination revealed a meniscal click. Arthroscopy of the stifle was performed with partial meniscectomy. The dog represented 2 years, 11 months following the original surgery for lameness and pyrexia. Radiographs were performed (Figure 2). Based on presentation and radiographs, septic arthritis was suspected and an arthrotomy was performed. On surgical exploration, septic arthritis was confirmed and mucopurulent discharge around the distal toggle implant was noted. The implant was removed, the joint was lavaged, and the implant was submitted for culture and sensitivity which revealed infection with E. coli. One month following removal, the patient represented for persistent left hindlimb lameness. Arthroscopy was performed and showed synovitis and fibrin deposition with no additional meniscal tears. No further follow up was noted.

### 2.2 Adaptation of the Clavien–Dindo system

The Clavien–Dindo classification system was adapted for application to veterinary orthopedic surgeries based off the human orthopedic adaptation by Sink et al.^8^ Like the modified system used in human orthopedics, the adapted system (aCD) consisted of five different grades based on the treatment required to treat the complication. Additional modifications for this study included subcategorization for grade V complications (death) to distinguish causes of death (secondary to the disease or because of euthanasia). Each of the five grades were further defined by providing specific examples of complications that would fall under each category (Table 1). Sequelae and failure to cure were defined and offered as possible alternative grading choices under the aCD scheme^4^, ^5^, ^6^, ^8^ These outcomes could be selected instead of a complication grade and would receive no aCD grade for comparison between the two systems.

**TABLE 1:** Adapted Clavien–Dindo postoperative complications grading system

Grade	Definition	Specific examples of complications
I	A complication that requires no treatment and has no clinical relevance; there is no deviation from routine follow‐up during the postoperative period; allowed therapeutic regimens include: antiemetics, appetite‐stimulants, anti‐inflammatories, analgesics, antibiotics, and physiotherapy	Postoperative fever, nausea, constipation, surgical wound problem not requiring a change in postoperative care, suture reactions, draining >48 h with no clinical ramification, skin irritation from clippers/surgical preparation, self‐resolving cough
II	A deviation from the normal postoperative course (including unplanned clinic visits) that requires outpatient treatment: either pharmacologic, close monitoring as an outpatient, or minor surgical procedures not requiring general anesthesia	Superficial wound infection either at incision site or attributable to external coaptation requiring additional clinic visits, transient neuropraxia from retraction, inflammation, anesthetic protocols (nerve blocking, epidurals), postoperative pain concerning enough to warrant additional clinical visits, delayed union requiring alteration in postoperative activity, more clinic visits for monitoring/radiographs or extended application of external coaptation, sedated migrating pin removal
III	A complication that is treatable but requires surgical, arthroscopic, or radiographic interventions, an unplanned hospital admission, or requires some type of management under anesthesia	Delayed or nonunion requiring addition of bone graft, unplanned implant disassembly/destabilization, or implant replacement/addition. Apparent or imminent implant failure requiring replacement and/or revision. Reluxation of a surgically treated luxated joint
IV	A complication that is life threatening, requires ICU admission for longer than what is typically warranted with postoperative recovery, is untreatable, and/or possesses the potential for permanent disability; a complication that requires organ resection	Osteomyelitis with systemic illness, or that requires hospitalization with vacuum, or open wound management. Fracture disease (nonunion/malunion) resulting in limb malalignment or myotendinous impairment with permanent alteration of ambulation. Necessity of revising any joint surgery or joint replacement surgery to an arthrodesis or excision arthroplasty. Failure of any surgery that results in limb amputation
Va	Death secondary to perioperative complication	
Vb	Euthanasia due to poor prognosis or owners' inability to manage the complication	
Sequelae	Events that are inherent and inevitable following the procedure	Bruising, erythema, swelling, scarring, joint laxity after splint bandage
Failure to cure	Failure of the original purpose of the surgery despite its successful execution	Bone/incision heals, but pain/lameness persists without discernible cause

Example of a possible sequelae: Male intact, bulldog – right cranial cruciate ligament rupture with associated stifle instability treated with TightRope stabilization; 2‐weeks post‐surgery the patient developed a mild seroma at the incision site. At the 2‐month recheck, the seroma had resolved with no evidence of discharge or drainage. Follow up phone calls with the owner revealed the patient was not lame and was pleased with the dog's recovery.

Example of a possible failure to cure: Female spayed, mixed breed dog – left cranial cruciate ligament rupture with associated stifle instability treated with TightRope stabilization; 7‐months post‐surgery the dog was persistently lame. Radiographs and palpation were performed under sedation which showed stable positioning of the implant, no radiographic abnormalities, and the stifle was stable on palpation with an absence of a meniscal click or crepitus. There was no further follow up.

Any case being graded by an assessor as possessing a sequela or representing a failure to cure was censored from the data set in keeping with the methodologies of Sink et al.^8^ to only compare case scenarios of true complications (*n* = 67). Additional comparative analysis was completed on the entire set of scenarios (true complications plus those determined to have sequelae or be failures to cure by at least one assessor) to determine the impact of including the two specific outcomes on reliability using the aCD system (*n* = 76).

### 2.3 Assessment of reliability

Four assessors evaluated the 76 scenarios. All four assessors were board certified diplomates of the American College of Veterinary Surgeons representing two different academic teaching hospitals. As the aCD system was new to all assessors, a training exercise was completed prior to the initiation of the study similar to that done by Sink et al.^8^ For this, each assessor was provided 10 training scenarios not included in the set of 76 cases. These example scenarios were graded with the aCD system and discussed as a group prior to the evaluation of the clinical scenarios. All assessors self‐described as being familiar with, and having clinically used, the Cook grading system and assessors were referred to the original manuscript^3^ for clarification regarding its application. The 76 clinical scenarios were distributed to the assessors through email as an attached document. There was no communication between assessors regarding the scenarios once they were distributed. The case scenario document possessed a designated space for the application of both the Cook and aCD system grades, or outcome measure. Assessors were given 2 months to grade all the scenarios.

### 2.4 Statistical analysis

Data analysis was performed using statistical software (SPSS Statistics for Macintosh, Version 26.0; IBM). Inter‐rater reliability assessment within the two methodologies was conducted using intraclass correlation coefficient (ICC) calculations and were reported with their associated 95% confidence intervals (CI) for each data set. The ICC ranged from 0 (no agreement) to 1 (perfect agreement). Intraclass correlation coefficient values of <0.5, 0.5 < *x* < 0.75, 0.75 < *x* < 0.9, and >0.9 were considered indicative of poor, moderate, good, and excellent reliability, respectively. Significance for all statistical tests was set at *p* < .05.

## 3 RESULTS

Seventy‐six case scenarios, from 68 dogs were evaluated and graded using both the aCD and Cook classification systems. When the complete set of 76 scenarios was analyzed, the Cook classification system had good interobserver reliability with an ICC of 0.848 (95% CI: 0.776–0.900), while the aCD system had moderate reliability with an ICC of 0.620 (95% CI: 0.459–0.742) (Table 2).

**TABLE 2:** Interobserver reliability assessment of the two grading methodologies for all 76 case scenarios

Grading method	ICC (76 cases)	95% CI	
		Lower bound	Upper bound
Cook	0.848	0.776	0.900
Clavien–Dindo	0.620	0.459	0.742

*Note*: Interobserver reliability assessment of the two grading methodologies for 76 scenarios in which complications and scenarios believed to represent either a sequela or failure to cure were included in the aCD grading system or given a categorical complication grade using the Cook system.

Abbreviations: ICC, intraclass correlation coefficient.

**FIGURE 3:**
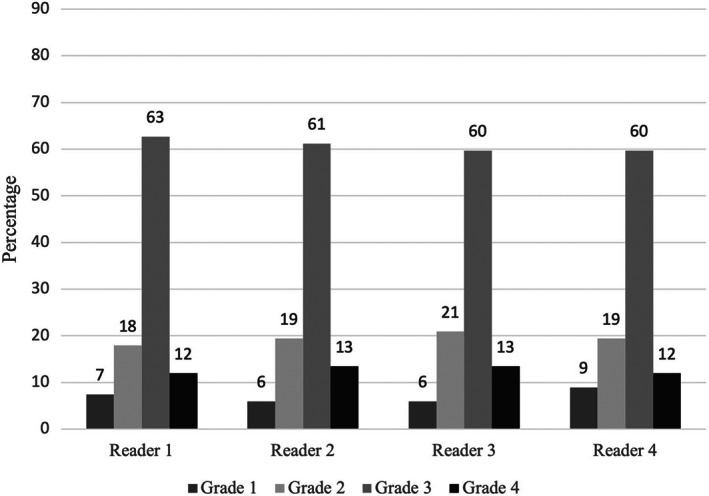
Distribution (%) of the 67 scenarios of true complications for each assessor using the adapted Clavien–Dindo system. No scenarios were marked as grade 5 complications.

**FIGURE 4:**
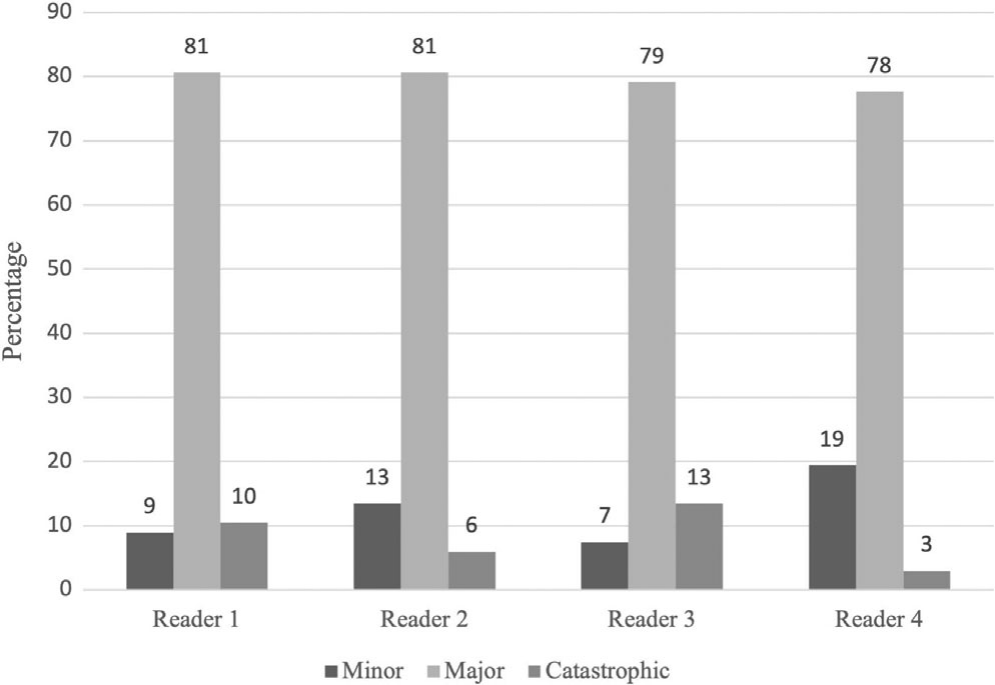
Distribution (%) of the 67 scenarios of true complications for each assessor using the Cook Classification system.

Using the aCD system, a total of nine scenarios were reported to be a sequelae (*n* = 3) or a failure to cure (*n* = 6) according to at least one assessor. These cases were censored from the overall set of scenarios resulting in 67 scenarios of true complications that underwent comparative analysis using the two grading systems. Of those 67 scenarios, grade 3 complications were the most reported by all assessors and constituted 60%–63% of all the scenarios when graded using the aCD system (Figure 3). No scenarios were graded as a 5. Using the Cook classification system, major complications were the most reported and constituted 78%–81% of all the scenarios (Figure 4). When censoring noncomplication outcomes (sequelae or failures to cure), the interobserver reliability of the aCD system was excellent with an ICC of 0.975 (95% CI: 0.964–0.984) and the reliability of the Cook system was good with an ICC of 0.857 (95% CI: 0.786–0.907) (Table 3).

**TABLE 3:** Interobserver reliability assessment of the two grading methodologies for 67 scenarios of true complications censoring noncomplication outcomes

Grading method	ICC (67 cases)	95% CI	
		Lower bound	Upper bound
Cook	0.857	0.786	0.907
Clavien–Dindo	0.975	0.964	0.984

*Note*: Interobserver reliability assessment of the two grading methodologies for 67 scenarios deemed to represent true complications in which scenarios possessing an outcome measure (sequela, failure to cure) were censored.

Abbreviations: ICC, intraclass correlation coefficient.

In general, there was disagreement between assessors in distinguishing noncomplication outcome sequelae cases from grade 1 aCD complications, and failure to cure outcomes from grades 1 and 2 aCD complications. In two of the three sequelae cases, more than one assessor scored the scenario as a grade 1 aCD complication. In five of the six failure to cure case scenarios, more than one assessor scored the scenario as a grade 2 complication, and in one of six cases more than one assessor scored the scenario as a grade 1 complication.

## 4 DISCUSSION

A reliable, standardized complication‐grading scheme is critical for an accurate assessment of postoperative complications and to allow for their comparison between studies. This study evaluated the application of an alternative postoperative grading system to a singular veterinary surgical procedure and compared its reliability to an existing classification system. Contrary to our hypothesis, differences were noted in the reliability of the two systems when evaluated between assessors at different institutions.

When all case scenarios, including those considered to be either a sequelae or a failure to cure, were graded and compared, the reliability of the aCD system was reduced compared to the Cook system. Despite the aCD offering more discrimination between specific types of complications, assessors were unable to consistently discern complications from noncomplication outcomes, sequelae and failures to cure. While these outcome terms have been accepted in human surgery, their novelty to veterinary surgeons may explain their less reliable application in this study. This was avoided in the Sink et al.^8^ study by prescreening cases prior to assessor evaluation so only scenarios including true complications were considered, and there was no option for an assessor to grade a case scenario as a sequelae or failure to cure. As Sink et al.^8^ and others emphasize, complications should be distinguished from sequelae, which are inevitable and inherent to the procedure, and failures to cure, which describe when the goal of surgery has not been achieved despite no clear adverse event associated with the procedure.^5^, ^8^ However, the inclusion of noncomplication cases to the sample population with associated expansion of the aCD system to seven possible choices (complication grades I–V plus two specific outcomes) reduced agreement.

Should future investigators choose to include these outcomes as alternatives to complication grades when classifying postoperative events, there could be predictable decreases in overall complication rates described for some procedures. For example, when examining complication types and incidence in cases undergoing tibial plateau leveling osteotomies (TPLO), Stauffer et al., reported an overall complication rate of 18.8%.^14^ Upon examination of their data, 9.3% of TPLO complications were recorded as being “short‐term complications” with 6% identified as possessing postoperative swelling (bruising or edema) which required no treatment.^14^ By definition of the aCD system,^4^, ^5^, ^6^, ^8^ many of these incidences could have been classified as a sequelae rather than a true complication, as postoperative bruising and swelling is an expected outcome following TPLO. Reclassifying these reported events as sequelae instead of complications would decrease the complication rate reported by Stauffer et al. Future studies could benefit from applying the aCD system and utilizing these novel terminology to report postoperative complications more accurately.

When grading scenarios of true complications, the aCD system was more reliable than the Cook system. While the Cook system has three main categories of complications, the aCD system has five. As the number of choices increases, there should be a reduction in interobserver reliability. However, using the aCD system, the interobserver reliability improved relative to the Cook system demonstrating that surgeons can discriminate between more choices of complication subtypes with higher reliability. There are many possible reasons for this finding. First, the aCD scoring system provides a more robust definition for each complication subtype, consistent with the methodologies of Sink et al.,^8^ including examples of each, than what is described by the manuscript by Cook et al.^3^ Improving and clarifying communication with standardized terminology with any grading system should improve consistency in application. Specific intent was made to avoid more subjective terminology when defining each subgrade of complication in keeping with the recommendations of Clavien and Dindo. Thus, a more complete definition using less subjective terminology of each complication grade likely reduced assessor ambiguity when distinguishing between complication types explaining the higher reliability even with more choices of complication types.

A major limitation of this study was that the sample population did not possess an even distribution of complication types to allow for the true validation of either the aCD or Cook classification systems. While this study did measure agreeability between assessors, conclusions regarding accuracy of either system cannot be made. Additionally, the study utilized a small number of assessors from only two different institutions, both within the United States. Additional multi‐institutional, multi‐national studies with even distributions of complications could assess both reliability and validity of the system for widespread use in veterinary orthopedics. Further, this study examined the application of complication grading systems involving a single orthopedic procedure. The decision to limit the study to a single procedure is in keeping with the work of Sink et al., who sought to demonstrate the Clavien–Dindo system could be adapted to orthopedic surgery as a proof‐of‐concept by initially examining only hip preservation surgery.^8^ Despite the initial training exercise, the inexperience of assessors in utilizing the novel aCD system of complication grading, in particular the use of noncomplication outcomes, may have negatively affected reliability. Due to the reported familiarity of all assessors to the Cook system, a group training and discussion exercise was not performed using the Cook system.

In conclusion, the adapted Clavien–Dindo grading system presented endeavors to achieve greater standardization, reliability, and nuance between subtypes in reporting complications and other noncomplication outcomes with less subjectivity. While this was achieved in events determined by all assessors as true complications, the aCD system proved less reliable in discriminating complications from sequelae and failure to cure. The Cook system demonstrated consistently good reliability in assessing postoperative complication grade although with less discernment between complications and sequelae and failure to cure. Further application of the aCD system and more detailed specifications of the terms sequelae and failure to cure in future studies may help to report true complication rates more accurately. This study demonstrates the potential utility of an alternative system for the grading of postoperative complications which offers more choice and discrimination between complication types but will depend on wider acceptance of novel terminology among veterinary surgeons to be reliably applied.

## ACKNOWLEGMENTS

Lisa Anderson, DVM for assistance in adapting the modified Clavien–Dindo System. Adrienne Siddens, CVT for assistance in reviewing the medical records. Elisabeth Fox, DVM for assistance in collecting radiographs associated with case scenarios.

## AUTHOR CONTRIBUTIONS

Barrett FM, BVM&S: study design, data collection, data analysis and interpretation, drafting and revising the manuscript, approval of the final article; Bleedorn JA, DVM, MS, DACVS‐SA: participation in grading of case scenarios, drafting and revising the manuscript, approval of the final article; Hutcheson KD, DVM, DACVS‐SA: participation in grading of case scenarios, drafting and revising the manuscript, approval of the final article; Torres BT, DVM, PhD, DACVS‐SA, DACVSMR: study design, participation in grading of case scenarios, data analysis and interpretation, statistical analysis of data, drafting and revising the manuscript, approval of the final article; Fox DB, DVM, PhD, DACVS: conceptualization of the study, participation in grading of case scenarios, data analysis and interpretation, drafting and revising the manuscript, approval of the final article.

## CONFLICT OF INTEREST

The authors declare no conflict of interest related to this report.
